# ᶫ-Leucine Loading and Release in MIL-100 Nanoparticles

**DOI:** 10.3390/ijms21249758

**Published:** 2020-12-21

**Authors:** Ivan E. Gorban, Mikhail A. Soldatov, Vera V. Butova, Pavel V. Medvedev, Olga A. Burachevskaya, Anna Belanova, Peter Zolotukhin, Alexander V. Soldatov

**Affiliations:** 1 The Smart Materials Research Institute, Southern Federal University, Sladkova Street 178/24, 344090 Rostov-on-Don, Russia; vbutova@sfedu.ru (V.V.B.); pmedvedev@sfedu.ru (P.V.M.); oburachevskaya@sfedu.ru (O.A.B.); anna.belanova@icloud.com (A.B.); soldatov@sfedu.ru (A.V.S.); 2 Research Laboratory “Biomedicine”, Southern Federal University, Stachki av. 194/1, 344090 Rostov-on-Don, Russia; pvzolotuhin@sfedu.ru

**Keywords:** MOF nanoparticles, MIL-100, targeted drug delivery, leucine, nanomedicine

## Abstract

Synthesis of the MIL-100 metal-organic framework particles was carried out by hydrothermal (HT) and microwave (MW)-assisted methods. Transmission electron microscopy showed formation of microparticles in the course of hydrothermal synthesis and nanoparticles for microwave-assisted synthesis. Powder X-ray diffraction confirmed formation of larger crystallites for hydrothermal synthesis. Particle aggregation in aqueous solution was observed by dynamic light scattering. However, the stability of both samples could be improved in acetic acid solution. Nitrogen sorption isotherms showed high porosity of the particles. ᶫ-leucine molecule was used as a model molecule for loading in the porous micro- and nanoparticles. Loading was estimated by FTIR spectroscopy and thermogravimetric analysis. UV-VIS spectroscopy quantified ᶫ-leucine release from the particles in aqueous solution. Cytotoxicity studies using the HeLa cell model showed that the original particles were somewhat toxic, but ᶫ-leucine loading ameliorated the toxic effects, likely due to signaling properties of the amino acid.

## 1. Introduction

Metal-organic frameworks (MOF) constitute a unique class of porous crystalline materials, where metal centers (metal ions or metal clusters) are connected by organic linkers into a three-dimensional lattice [1,2,3,4]. Since a number of different combinations of their structural elements can be used to create MOFs, the lattice parameters and chemical properties of these materials can also be tuned for specific applications. More than 20,000 variations of MOFs are known at the moment [5,6], and their pores could reach 98 Å in diameter [7] and occupy more than 50% of the volume. The surface area varies from 1000 to 10,000 m^2^/g [8], and the density could reach 0.13 g/m^3^ [9]. Depending on the structure, composition, and functionalization, MOFs could have unique sensory [10], sorption [11], catalytic [12], and other properties. That is why MOFs application in industry is developing at a record-breaking pace nowadays. Moreover, these materials are quite promising candidates for targeted drug delivery and controlled drug release.

Depending on the structure, MOF targeted drug delivery could be performed in different ways [13,14]. One option is the decomposition of MOF into biocompatible and bioactive components. Such an approach was used in Zn azelate MOF, which was composed of antibacterial and dermatologically active components [15]. Another approach is based on the storage of biologically active agents inside the MOF pores. It is possible to tune the MOFs structure to enable control over drug release. The latter makes it possible to perform a controlled or prolonged drug release from MOF particles.

MIL-100 is composed of metal clusters connected by trimesic acid. Its high surface area, large pore sizes (5.5 and 8.6 Å), and its biocompatible composition make it a suitable material for drug delivery. The original synthesis includes metallic iron, trimesic, nitric, and hydrofluoric acids [16]. All components were mixed into an autoclave and heated to 200 degrees Celsius for 24 h. The article also presents the geometric structure analysis of the resulting substance and determines the pore size. Nevertheless, due to nitric and hydrofluoric acids, this method is not environmentally friendly, and its large-scale production could be harmful. This problem was solved in 2012 when a green method for the synthesis of MIL-100 (Fe/Cr/Al) was proposed [17]. The microwave-assisted method reduces the synthesis time from 24 h to 6 min. Following the green chemistry route, no nitric and hydrofluoric acids were used. Iron chloride was also used instead of metallic iron. Studies on MIL-100 nanoparticle behavior in vivo showed that the sample was removed from the bloodstream, accumulated in the liver, and was gradually excreted [18]. Doxycycline monohydrate (DOX) and tetracycline hydrochloride were loaded into the pores of MIL-100 by means of post synthetic exchange of activated MIL-100 particles in ethanol solution [19]. Authors estimated a 25% increase in drug release compared to traditional methods.

However, information regarding molecule positioning inside the pores of MIL-100 is rarely available. Leucine could be used as a model molecule to study the positioning of the molecule in the pore of MIL-100, due to different functional groups (carboxyl, methoxy, and amine), which could govern the positioning of leucine in the structure of MIL-100.

Moreover, growing evidence shows complex roles for ᶫ-leucine in regulating protein and lipid metabolism in animals, in addition to being a building block for protein synthesis. ᶫ-leucine activates the mammalian target of the rapamycin (mTOR) signaling pathway, resulting in enhanced cellular respiration and energy partitioning [20]. Moreover, hypoxic signaling through mTOR results in significant changes in gene expression and cellular behavior in hypoxic cells that could modulate tumor growth [21]. Prolonged release of ᶫ-leucine could be achieved using porous MOF-based nanocomposites. Moreover, the role of ᶫ-leucine as a trigger for mTOR renders it a significant player in the PI3K/Akt signaling pathway as well. These properties of ᶫ-leucine suggest it is underestimated in biomedicine. However, how ᶫ-leucine should be administered for maximum efficacy of the respective approaches in ageing management, cosmetics, rehabilitation, and nutrition is an open question. One possible answer is using next-generation nanocarriers (such as MOFs) for targeted, concentrated, or prolonged delivery via an oral route.

## 2. Results and Discussion

**Figure 1:**
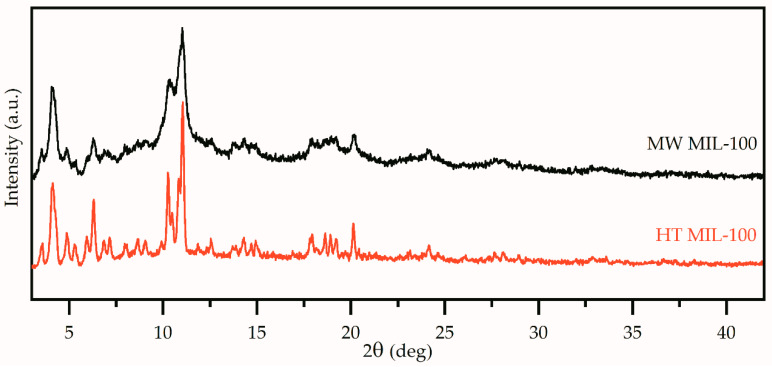
PXRD patterns of the MIL-100 (Fe) synthesized by microwave-assisted (black) and hydrothermal (red) methods.

The PXRD patterns of the MIL-100 (Fe) synthesized by means of microwave-assisted and hydrothermal methods are illustrated in Figure 1. The peak positions and relative intensities of collected patterns were in agreement with the results reported for MIL-100 (Fe) [16]. The peak widths for the HT MIL-100 pattern were evidently smaller compared to those of MW MIL-100. This could be attributed to the larger sizes of the crystallites. Such results are quite expected as hydrothermal synthesis usually results in larger particles with improved crystallinity compared to the microwave-assisted method. The microwave method usually requires less time, however, and results in smaller particles with weaker crystallinity.

**Figure 2:**
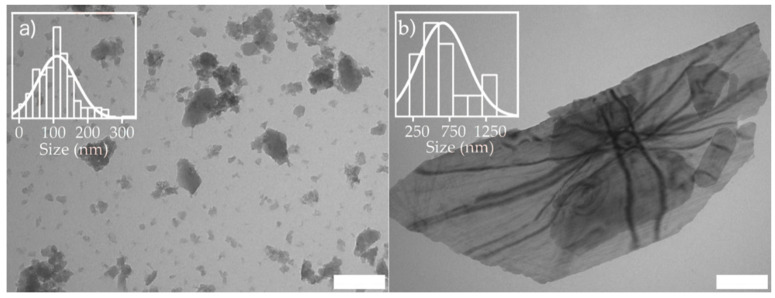
Transmission electron microscopy images and distribution analysis of MIL-100 (Fe) particles synthesized by microwave-assisted (**a**) and hydrothermal (**b**) methods. The scale bar is 200 nm. The insets show size distribution.

Transmission electron microscopy images are presented in Figure 2. A larger aggregate of MW MIL-100 particles was observed on the TEM image. There was also an evident difference in the size of the particles for MW MIL-100 samples compared to HT MIL-100. Expectedly, this supports the formation of small particles of MW MIL-100 and large for HT MIL-100. Analysis of the TEM images gives a rough estimation of 110 ± 60 nm for the MW MIL-100 sample.

However, the analysis of size distribution of HT MIL-100 particles did not show unimodal distribution. There was a fraction of smaller particles with a mean size of about 500 nm. Large micrometer particles were also observed for HT MIL-100. Interestingly, transparent TEM images of HT MIL-100 particles (Figure 2b) suggest the nanosheet structure of the particles. Moreover, Kikuchi lines were clearly visible on TEM images of the large crystal, suggesting bending of the nanosheet. This bending may occur due to the presence of smaller particles, which were also visible under the largest one. The reason for the deviation of particle sizes from a normal distribution for the HT MIL-100 sample could be the crumpling of the nanosheets of MIL-100.

**Figure 3:**
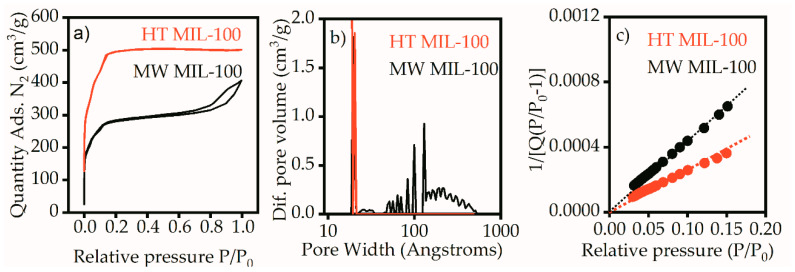
Nitrogen adsorption–desorption (**a**) isotherms, (**b**) pore size distribution, and (**c**) BET equation graph.

The shape of the isotherm for the HT MIL-100 sample (Figure 3a) corresponds to type I, according to the IUPAC classification, and indicates microporous material. For this sample, the adsorption and desorption branches merged, which indicates the absence of meso pores. This was also confirmed by the pore size distribution, on which the sample showed one peak in the region of 2 nm (Figure 3b).

The isotherm for MW MIL-100 is of type IV, according to the IUPAC classification (Figure 3a). The isothermal region under the relative pressures 0–0.2 corresponded to the formation of a monolayer and indicated the presence of micropores. However, a pronounced hysteresis loop caused by the effect of capillary condensation indicated the presence of mesopores. These data are in good agreement with the pore size distribution on Figure 3b. Along with micropores in the 2 nm region, MW MIL-100 had intense peaks corresponding to the pores larger than 10 nm. The specific surface areas for both samples were calculated by the Brunauer–Emmett–Teller (BET) method in the range of relative pressures of 0.03–0.15 at 25 points (Figure 3c). The correlation coefficient was not less than 0.9996, which shows good correspondence of the selected range to the linear form of the BET equation. The specific surface areas were 1929 m^2^/g for the HT MIL-100 and 1075 m^2^/g for the MW MIL-100. The available pore volumes calculated at the point P/P_0_ = 0.97 were 0.77 for HT MIL-100 and 0.60 for MW MIL-100. A typical specific surface area of bulk MIL-100 (Fe) is usually about 2800 m^2^/g [16]. Such reduction in the surface area could be the result of aggregation of MW MIL-100 nanoparticles and crumpling of HT MIL-100 nanosheets observed on TEM images.

**Figure 4:**
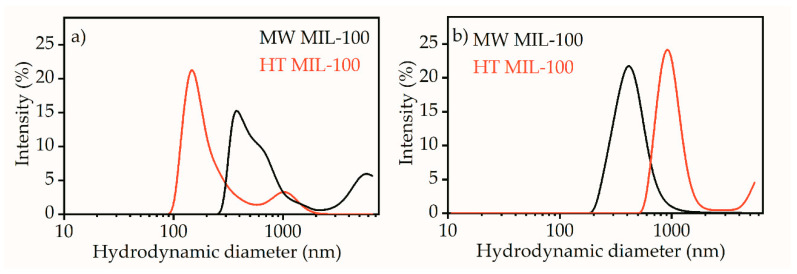
Hydrodynamic size estimated by dynamic light scattering for MW (black) and HT (red) MIL-100 samples in water (**a**) and acidic acid (**b**) solutions. Hydrodynamic diameter is presented in logarithmic scale.

The hydrodynamic diameter of MW MIL-100 particles showed a bimodal distribution, with the main peak at about 450 nm and a shoulder at micrometer sizes (Figure 4a). The latter could be a signature of particle aggregation into large clusters. A similar trend was observed for HT MIL-100; however, the main peak was at 150 nm, and a large diameter shoulder was at about 900 nm. Surprisingly, the large area nanosheets of HT MIL-100 showed a small hydrodynamic radius. Acetic conditions could prevent formation of large aggregates of MOF particles. Figure 4b shows the hydrodynamic diameter distribution of the particles dispersed in a 50% solution of acetic acid. The mean hydrodynamic diameter of MW MIL-100 in acidic conditions was roughly 430 nm. That is in good agreement with the hydrodynamic size of the same particles in aqueous solution. However, in acidic conditions there is no contribution from large aggregates. This suggests that in both water and acidic acid solution the hydrodynamic size of MW MIL-100 particles is on the order of 400–500 nm. The size distribution of HT MIL-100 showed a bimodal distribution with the maximums at about 850 nm and 5500 nm. The first value was in a good agreement with statistics from TEM measurements. Although evident contributions from micrometer aggregates arise in neutral aqueous solution, it should be stated that the major fraction of the particles was in the nanometer range.

**Figure 5:**
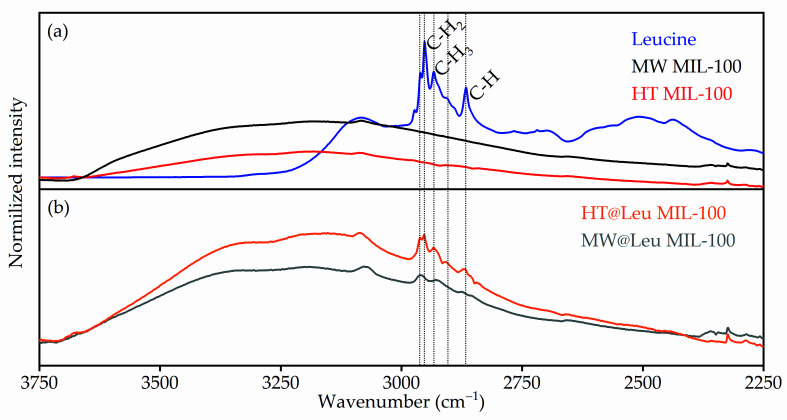
(**a**) FTIR spectra of pure HT MIL-100 (red), MW MIL-100 (black), and ᶫ-leucine (blue). (**b**) FTIR spectra of loaded HT@Leu MIL-100 (orange), MW@Leu MIL-100 (grey).

**Figure 6:**
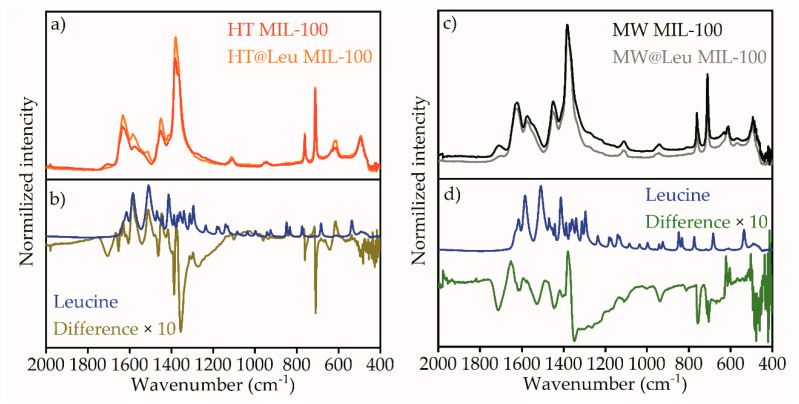
Fingerprint region of FTIR spectra for (**a**) MW MIL-100 and MW@Leu MIL-100 compared to (**b**) leucine and difference spectrum for MW@Leu MIL-100 minus MW MIL-100. Fingerprint region of FTIR spectra for (**c**) HT MIL-100 and HT@Leu MIL-100 compared to (**d**) leucine and difference spectrum for HT@Leu MIL-100 minus HT MIL-100.

The loading of ᶫ-leucine to the MIL-100 containers was performed using post synthetic exchange in neutral aqueous solution. FTIR spectroscopy was used to control ᶫ-leucine loading to the MIL-100 nanocontainers (Figure 5). The collected FTIR spectra were in good agreement with published data [22,23]. In addition, peak 3085 cm^−1^ corresponding to C-H oscillations was observed without changes in both spectra. In the fingerprint region located from 2000 to 500 cm^−1^ (Figure 6a,c), peak positions correspond to chemical bonds in MIL-100. Mostly, peaks in this region correspond to linker vibrations, as, for example, 1721 cm^−1^ corresponds to C=O, and 1627 cm^−1^ ν(C-O). Symmetric and asymmetric vibrations of the carboxyl group were located at 1447 cm^−1^ and 1375 cm^−1^. Next, vibrations of the benzene ring corresponded to 1108 cm^−1^ and 946 cm^−1^, and C-H vibrations corresponded to the benzene ring 750 cm^−1^ and 790 cm^−1^. ᶫ-leucine on the FTIR spectrum was clearly visible in the water region (3750–2250 cm^−1^) in Figure 5 and fingerprint region (2000–400 cm^−1^).

Figure 5a shows the FTIR spectra of pure MW MIL-100 and HT MIL-100 samples compared to those of leucine. It can be estimated that the intensity of the water peak in samples loaded with ᶫ-leucine was significantly lower (Figure 5b) than that in pure samples. This suggests that the presence of ᶫ-leucine in the pores of a metal-organic framework structure displaces a certain number of water molecules. The presence of ᶫ-leucine confirms an increase in the intensity of the peaks corresponding to vibrations of the C-H (2867 cm^−1^), C-H_3_ (2904 and 2934 cm^−1^), and C-H_2_ (2952 and 2961 cm^−1^) groups of ᶫ-leucine.

To determine the presence of leucine in loaded samples, the fingerprint region FTIR spectra were analyzed in Figure 6. The spectra of HT MIL-100 is compared to those of HT@Leu MIL-100 in Figure 6a. Figure 6b shows the difference spectrum of leucine-loaded and pure HT MIL-100 compared to the spectrum of pure leucine. Difference spectrum reveals the increase in the intensity of peaks corresponding to the leucine spectrum, which was located in the region of vibrations of the carboxyl group (1584, 1513, 1413 cm^−1^). These changes were also present on the spectrum for the sample loaded with leucine. The difference spectrum also showed the coincidence of the intensity increase with the spectrum of ᶫ-leucine in the regions of 1294 and 839 cm^−1^. Nevertheless, these peaks were difficult to determine on the spectrum of MIL-100 loaded with ᶫ-leucine due to their low intensity in relation to the original MIL-100 peaks.

Figure 6c shows FTIR spectra of pure MW MIL-100 and MW@Leu MIL-100 loaded with ᶫ-leucine. Figure 6d shows their difference spectrum compared to the spectrum of pure leucine. Similar changes were observed when ᶫ-leucine was loaded with the HT MIL-100 sample, but to a much lesser extent. Thus, it can be concluded that absorption of ᶫ-leucine was less efficient.

**Figure 7:**
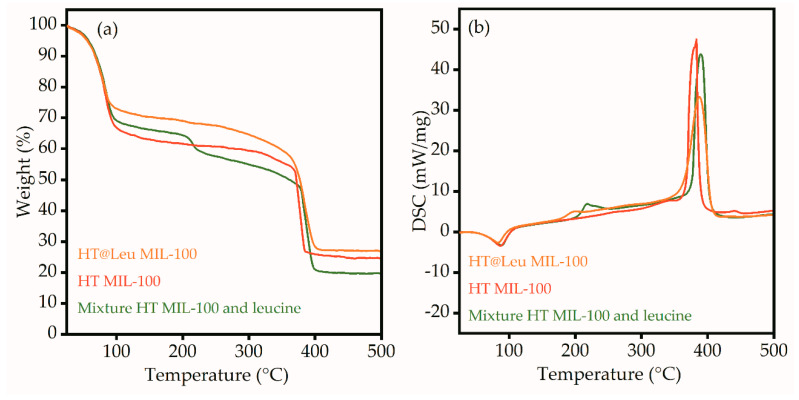
TGA (**a**) and DSC (**b**) analyses of pure MIL-100, MIL-100 loaded with ᶫ-leucine, and mixture of MIL-100 and ᶫ-leucine.

Figure 7 shows the thermogravimetric analysis and differential scanning calorimetry (TGA-DSC) of the HT MIL-100, HT@Leu MIL-100, and mixture of pure leucine and HT MIL-100. All samples lost weight at 75 °C (Figure 7a). This is attributed to the evaporation of water molecules from the surface and pores of the particles, and additionally confirmed by the endothermicity of the process (Figure 7b). Paying attention to the TGA curve of the mechanical mixture of leucine and HT MIL-100 in Figure 7a, one can see the weight loss at a temperature of 218 °C and the corresponding exothermic process on the DSC profile (Figure 7b). This temperature corresponds to the degradation temperature of leucine. A similar process can be seen on the DSC profile of the HT@Leu MIL-100. However, the peak of exothermic process shifted to the lower temperatures. The weight loss on the TGA curve was also much lower. This aspect can be explained by the process of destruction of ᶫ-leucine in the pores of the MIL-100; however, the change in mass was small and, therefore, not reflected in the TGA curve. The last endothermic process was associated with the decomposition of the MIL-100 framework. The difference in the intensity of this peak on the DSC profile is explained by the different weight loading of the samples.

**Figure 8:**
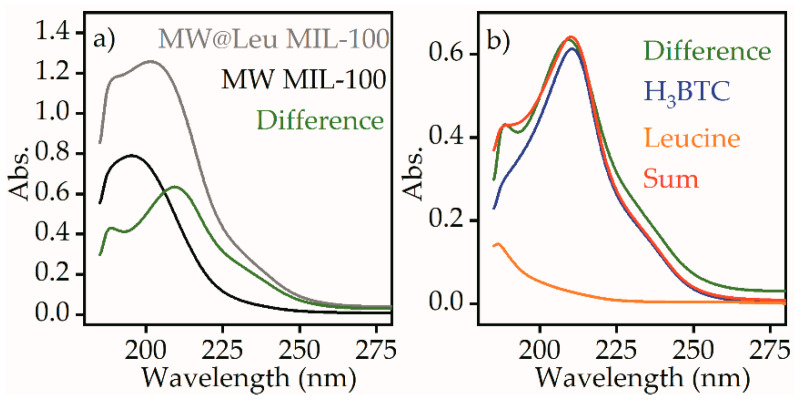
UV-VIS spectra measured for the diluted supernatant of MW@Leu MIL-100 (grey) and MW MIL-100 (black) samples and their difference spectrum (green) (**a**). Difference spectra (green), leucine (orange), H_3_BTC (blue), and a sum of leucine and H_3_BTC components (red) (**b**).

The release of the leucine from the MW@Leu MIL-100 sample was quantified using UV-VIS spectroscopy. Figure 8a shows the spectra of the supernatant for MW@Leu MIL-100 and MW MIL-100 samples. The difference spectrum shows the traces of the molecules that were present in the supernatant of the leucine-loaded sample with higher concentration compared to the pure sample.

The difference spectrum did not correspond to a pure leucine component, which has an absorption peak at 187 nm. However, the difference spectrum could be reproduced by a linear fit combination of leucine and H_3_BTC component, which is one of the components of MIL-100 structure. Such a linear fit combination suggests 29 µg⋅mL^−1^ concentration of H_3_BTC and 17 mg⋅mL^−1^ concentration of leucine after release.

The results of cytotoxicity tests are shown in Table 1.

One of the main, but not the most significant, results of the study was the demonstration of marked toxicity of the MIL-100 in its present form. These data suggest the nanocarrier should be modified or re-designed. However, the main outcome of the study from a biological point of view was ᶫ-leucine-loaded MIL-100 showing significantly less cytotoxicity compared to the unloaded counterpart. This pointed out an important phenomenon: cell physiology improvement and chemical toxicity amelioration by a common (even if essential) amino acid ᶫ-leucine. Although ᶫ-leucine is a basic amino acid, and despite the well-known fact of its ability to modulate one of the central signaling systems of the cell (the mTOR/Akt sub-pathway of the PI3K/Akt pathway), ᶫ-leucine-based biomedical technology is far from well-developed. In our study, however, we demonstrate that ᶫ-leucine may serve as a potential candidate for targeted detoxification therapy and the related approaches in medicine, cosmetics, rehabilitation, and so on.

At the same time, the results clearly demonstrate that using basic metabolites for re-wiring cellular signaling strongly depends on targeted delivery. The reasoning is as follows. In our experiments, the cells were incubated under standard conditions—in DMEM medium supplemented with 10% fetal bovine serum. This culturing system contains ᶫ-leucine as a standard component, at a concentration of about 100 μg/mL or higher. On the other hand, the MOF-loaded ᶫ-leucine, according to our estimates, only accounted for less than 5% of total ᶫ-leucine in cell culture medium. However, this small fraction, when introduced to the cells by the nanocarrier delivery, resulted in emergent changes in the cellular reaction to the initially toxic nanocarrier. Accordingly, concentrated and targeted ᶫ-leucine (and other similar signaling-modulating small molecules) administration may serve as a powerful tool in signaling ‘re-wiring’ biomedical approaches in detoxification therapy, ageing management, cosmetics, rehabilitation, nutrition, and so on.

## 3. Materials and Methods

### 3.1. Synthesis

All reagents were purchased from Sigma-Aldrich (St. Louis, MO, USA) and used without further purification. Ultrapure water (18 MΩ·cm) was produced by Simplicity UV (Millipore, Nihon Millipore, Tokyo, Japan).

Hydrothermal synthesis (HT MIL-100): MIL-100 (Fe) particles were synthesized by means of a modified hydrothermal method [18]. For hydrothermal synthesis, 0.336 g of Fe powder and 0.84 g of 1,3,5-H_3_BTC were added to 30 mL of ultrapure water and mixed for 30 min. Then, 0.2 mL of HF and 0.15 mL of HNO_3_ were added. The resulting suspension was transferred to a 100 mL Teflon autoclave (Berghof BR-100, Eningen, Germany) and heated at 150 °C for 20 h. Finally, the resulting orange solid was washed several times with water, centrifuged, and dried in a vacuum oven overnight.

Microwave-assisted synthesis (MW MIL-100): MIL-100 (Fe) particles were prepared by a microwave-assisted method [19]. Briefly, 2.43 g of iron (III) chloride hexahydrate and 0.84 g of 1,3,5-H_3_BTC were dissolved in ultrapure water (30 mL). The suspension was heated to 130 °C for 10 min, then maintained at this temperature for 5 min. After the synthesis, the sample was washed and dried using the standard procedure mentioned above.

### 3.2. ᶫ-Leucine Loading and Release

ᶫ-leucine loading into the pores of the MIL-100 particles was carried out using post synthetic exchange. Both samples (0.2 g each) were kept in a muffle furnace SNOL 8.2/1100 (AB Umega, Ukmergė, Lithuania) at 120 °C for 1 h for activation. Then, the activated material was added while stirring to 15 mL of 0.2 mmol aqueous solution of ᶫ-leucine. The resulting mixture was stirred for 16 h and then was washed with clean water and dried. ᶫ-leucine loaded HT and MW MIL-100 samples are denoted as HT@Leu MIL-100 and MW@Leu MIL-100, respectively.

To study the release of ᶫ-leucine from both HT@Leu and MW@Leu MIL-100, samples modified with ᶫ-leucine as well as control samples that did not undergo post synthetic processing were stirred in 4 mL of ultrapure water for 2 h. For each sample, the supernatant was taken after centrifugation and diluted tenfold with ultrapure water in order to enable UV-VIS measurements in 10 mm quartz cells with reasonable absorption.

### 3.3. Characterization

The powder X-ray diffraction (PXRD) patterns were collected on a Bruker D2 PHASER diffractometer (Billerica, MA, USA) using CuK*α* radiation (*λ* = 0.1541 nm). Transmission electron microscopy (TEM) images were collected using a Tecnai G2 BioTwin (FEI Company, Hillsboro, OR, USA) microscope operated at 120 kV. The FTIR spectra were collected on a Bruker VERTEX 70 spectrometer (Billerica, MA, USA) in transmission geometry at room temperature. Thermogravimetric analysis (TGA) and differential scanning calorimetry (DSC) were measured using a STA 449 F5 Jupiter (NETZSCH-Gerätebau GmbH, Selb, Germany) in the air atmosphere. The hydrodynamic diameters of the particles were analyzed using a dynamic light scattering NANO-Flex (Microtrac, Montgomeryville and York, PA, USA), equipped with 780 nm laser. Prior to the measurements, the samples were dispersed in pure water and acidic aqueous solutions. Nitrogen adsorption isotherms were measured using an ASAP 2020 (Micrometrics, Norcross, GA, USA) porosimetry system. Before the measurement, the samples were degassed at 100 °C under dynamic vacuum for 24 h. Nitrogen adsorption was measured at −196 °C. The pore size distribution was calculated according to the model of slit-like pores by the density functional theory method based on data obtained for the entire adsorption isotherm.

A double-beam UV2600 spectrometer (Shimadzu, Kyoto, Japan) equipped with 10 mm quartz cells was used in transmission geometry to monitor the release of ᶫ-leucine in aqueous solution. A calibration curve was used to quantify the concentration of ᶫ-leucine in the aqueous solution.

### 3.4. Toxicity Studies

HeLa cells were used as a model for testing cytotoxicity in vitro. The cells were seeded in 24-well plates (SPL Life Sciences, Gyeonggi-do, Korea) in a GlutaMax DMEM culture medium (Thermo Fisher Scientific, Waltham, MA, USA) supplemented with 10% fetal bovine serum (GE Healthcare, Boston, MA, USA). The cells were cultured at 37 °C and 5% CO_2_ with passive wetting in a Sanyo 180-MC incubator. To determine toxicity, nanoagents were resuspended in saline. Nanoagents and the carrier (as a control solution) were introduced into the culture medium at a final concentration of 50 μg/mL. Then, the cells were incubated for 24 h, after which the analysis of cell viability was carried out by the exclusion of trypan blue. Briefly, the cell culture supernatant was collected and centrifuged to collect the detached dead cells, while the cell layer was detached with 0.25% trypsin-EDTA solution (PanEco, Moscow, Russia), and centrifugation of the detached cells followed. The supernatant dead cells pellet and the detached cells pellet were combined, brought to 100 µL with 1× DPBS (Merck, Germany), and re-suspended. Then, 20 µL of the suspension was combined with 20 µL of 0.4% trypan blue (Thermo-Fisher Scientific, Waltham, MA, USA). After the recommended 2 min incubation, the cells were analyzed using an automatic cell viability analyzer Countess II FL (Thermo-Fischer Scientific, Waltham, MA, USA). Each bioreplicate was analyzed in two technical replicates, followed by averaging the technical replicates. The data were obtained as a result of three independent experiments conducted on different days. In total, there were nine samples in each of the groups. Solutions of samples were prepared at a concentration of 5 mg/mL in 0.9 % NaCl. The samples were limited to suspension and precipitated after a while. Therefore, before being introduced into the culture, they were treated with ultrasound. The final concentration of nanocontainers based on MIL-100 was 50 μg/mL.

## 4. Conclusions

Synthesis of MIL-100 particles of various sizes was carried out using microwave-assisted and hydrothermal methods. The size of the particles was analyzed using transmission electron microscopy and dynamic light scattering. The size of the MIL-100 particles synthesized by the solvothermal method had a unimodal distribution, and rough estimation of the mean size of the main fraction suggested 500 nm; however, a contribution of micrometer particles was also evident. TEM images also suggest formation of the particles with flat nanosheet morphology. The size of the MIL-100 particles synthesized by the microwave-assisted method was about 110 nm. Post synthetic loading of ᶫ-leucine in an aqueous medium led to leucine deposition inside the pores of the framework. ᶫ-leucine loading was confirmed by FTIR spectroscopy using C-H (2867 cm^−1^), C-H_3_ (2904 and 2934 cm^−1^), and C-H_2_ (2952 and 2961 cm^−1^) peaks of leucine. Characteristic peaks of ᶫ-leucine were observed in different FTIR spectra of loaded vs pure particles, which supports successful loading of leucine. TGA and DSC analyses also confirmed leucine loading inside the pores of MIL-100. UV-VIS spectroscopy was used to quantify the leucine release in aqueous solution. For the obtained samples, cytotoxicity studies were conducted. The MIL-100 particles loaded with ᶫ-leucine showed a 4% decrease in relation to the control study, while the pure MIL-100 showed a 17% decrease. MIL-100 nanoparticles loaded with leucine have the potential for biomedical applications due to reduced toxicity.

## Figures and Tables

**Table 1:** Results of cytotoxicity tests.

Group	Survival, Median [25th, …, 75th Percentiles]	Mean ± Standard Deviation	Mann–Whitney p-Level, Compared to the Control Group	Percent Change Compared to the Control Group
Control	100.00 [96.75, …, 100.00]	98.61 ± 2.23	-	-
MW MIL-100	83.00 [56.75, …, 91.50]	76.78 ± 20.11	<0.001	↓ 17%
MW@Leu MIL-100	96.00 [81.75, …, 97.75]	90.78 ± 9.52	0.019	↓ 4%
